# Identifying predictive features of autism spectrum disorders in a clinical sample of adolescents and adults using machine learning

**DOI:** 10.1038/s41598-020-61607-w

**Published:** 2020-03-18

**Authors:** Charlotte Küpper, Sanna Stroth, Nicole Wolff, Florian Hauck, Natalia Kliewer, Tanja Schad-Hansjosten, Inge Kamp-Becker, Luise Poustka, Veit Roessner, Katharina Schultebraucks, Stefan Roepke

**Affiliations:** 1 Department of Psychiatry, Charité - Universitätsmedizin Berlin, Campus Benjamin Franklin, Berlin, Germany; 2 Department of Child and Adolescent Psychiatry, Psychosomatics and Psychotherapy, Philipps University, Marburg, Germany; 3 Department of Child and Adolescent Psychiatry, TU Dresden, Dresden, Germany; 4 Department of Information Systems, Freie Universität Berlin, Berlin, Germany; 5 Department of Child and Adolescent Psychiatry and Psychotherapy, Central Institute of Mental Health, Medical Faculty Mannheim/University of Heidelberg, Mannheim, Germany; 6 Department of Child and Adolescent Psychiatry, University Medical Center, Göttingen, Germany; 7 Department of Psychiatry, New York University School of Medicine, New York, USA; 8 Vagelos School of Physicians and Surgeons, Department of Emergency Medicine, Columbia University Irving Medical Center, New York, USA

**Keywords:** Human behaviour, Autism spectrum disorders

## Abstract

Diagnosing autism spectrum disorders (ASD) is a complicated, time-consuming process which is particularly challenging in older individuals. One of the most widely used behavioral diagnostic tools is the Autism Diagnostic Observation Schedule (ADOS). Previous work using machine learning techniques suggested that ASD detection in children can be achieved with substantially fewer items than the original ADOS. Here, we expand on this work with a specific focus on adolescents and adults as assessed with the ADOS Module 4. We used a machine learning algorithm (support vector machine) to examine whether ASD detection can be improved by identifying a subset of behavioral features from the ADOS Module 4 in a routine clinical sample of N = 673 high-functioning adolescents and adults with ASD (n = 385) and individuals with suspected ASD but other best-estimate or no psychiatric diagnoses (n = 288). We identified reduced subsets of 5 behavioral features for the whole sample as well as age subgroups (adolescents vs. adults) that showed good specificity and sensitivity and reached performance close to that of the existing ADOS algorithm and the full ADOS, with no significant differences in overall performance. These results may help to improve the complicated diagnostic process of ASD by encouraging future efforts to develop novel diagnostic instruments for ASD detection based on the identified constructs as well as aiding clinicians in the difficult question of differential diagnosis.

## Introduction

Autism Spectrum Disorders (ASD) comprise a range of pervasive neurodevelopmental disorders with a population prevalence of approximately 1%^1^. They are characterized by early-onset persistent impairments in social communication and interaction as well as the presence of restricted, repetitive behaviors or interests^2,3^. Diagnosing ASD is a complicated, lengthy and time-consuming process, which requires outstanding and specific clinical expertise^4,5^. Although research makes constant progress in understanding the underlying genetic and neurobiological factors associated with ASD, there are currently no reliable biological markers for ASD and the diagnosis remains based on behavioral symptoms^1,6,7^. The current so-called “gold standard” of ASD diagnosis comprises the use of various standardized diagnostic instruments that assist clinicians in reaching a best-estimate clinical diagnosis^7–9^. Two of the most widely used diagnostic instruments are the Autism Diagnostic Observation Schedule (ADOS respectively ADOS-2 for the revised second edition)^10,11^ and the Autism Diagnostic Interview – Revised (ADI-R)^12^. The ADI-R is a semi-structured interview administered to parents or caregivers that focuses on developmental history and current symptom presentation. The ADOS is a standardized semi-structured diagnostic observation scale designed to assess important social-communicative behaviors as well as stereotypic and repetitive behavioral features. The ADOS includes four different modules for different age and language levels, with Module 4 intended for verbally fluent adolescents and adults. For each module, there is a diagnostic algorithm that allows for the classification of ASD or non-ASD^10,11,13,14^. The ADOS is one of the psychometrically best-evaluated diagnostic tools in ASD, especially in children (Modules 1 and 2)^4,15–17^, with fewer studies investigating the ADOS in adolescents and adults (Modules 3 and 4)^18–21^. Although a good psychometric quality has been widely demonstrated, existing studies suggest a lower diagnostic utility in naturalistic clinical settings as well as with older individuals^8,22–24^. While most cases with ASD are diagnosed in childhood, the diagnosis oftentimes remains unnoticed until adolescence and adulthood, particularly in those individuals with at least average cognitive and language abilities, better adaptive functions and more subtle symptom presentations^25,26^. Diagnosing ASD in high-functioning adolescents and adults (i.e. without intellectual disability) can be even more challenging compared to childhood due to various factors: a care-giver based developmental history as acquired with the ADI-R is oftentimes unavailable in older individuals^25^ and the individual’s self-report of symptoms may be diminished due to impaired self-referential cognition^27^. Additionally, learned compensatory skills might conceal impairments, thereby reducing diagnostic accuracy of observational tools such as the ADOS^25^. Furthermore, most adults with ASD have at least one comorbid psychiatric disorder^1,28^ and ASD symptoms frequently overlap with those of other psychiatric conditions^19,23^ thereby further complicating differential diagnosis. Nonetheless, establishing an accurate and timely diagnosis is of great importance for those affected and the planning of suitable psychosocial interventions in order to promote positive outcome^29–31^.

Previous studies have applied machine learning techniques to examine whether the process of diagnosing ASD can be improved by statistically identifying reduced subsets of features from existing diagnostic instruments reaching from self-administered screening questionnaires to clinician-administered diagnostic tools (for a recent overview, see Thabtah^32^). A few authors have shown that efficiency and accessibility of existing pre-diagnostic screening questionnaires such as the Autism-Spectrum Quotient (AQ)^33–35^ or the Social Responsiveness Scale (SRS)^36,37^ can be improved using machine learning. Similar machine learning experiments have been run to identify subsets of behavioral features from clinician-administered diagnostic tools, namely ADOS (Module 1 to 3)^38–42^ and ADI-R^36,39,43^. Findings of these studies suggest that ASD detection in children can be achieved with substantially fewer items compared to the original ADOS and ADI-R algorithms while retaining high diagnostic accuracy, sensitivity and specificity. To our knowledge, this has not yet been examined in a sample of adolescents and adults as assessed with the ADOS Module 4. The purpose of the present study therefore was to expand on the existing literature with a specific focus on high-functioning adolescents and adults. Advances of the present study are a large and balanced routine clinical sample of adolescents and adults with best-estimate clinical diagnoses of ASD and relevant psychiatric differential diagnoses, thereby being a good representation of the actual population presenting to ASD assessment settings. We aim to identify the diagnostically most informative features from the ADOS Module 4 that accurately differentiate between individuals with ASD and individuals with other clinically complex presentations using a data-driven machine learning approach. Although all items of the ADOS focus on relevant behavioral concepts, some items may be more discriminative and have higher classification ability particularly in a challenging clinical sample of older individuals that were all initially suspected of ASD. Identifying essential subsets of behavioral features that distinguish ASD from non-ASD cases could contribute to an enhancement of the complex diagnostic process in multiple ways: to improve existing diagnostic tools (i.e. revise existing classification algorithms), to shorten existing diagnostic tools such as the ADOS (for a critical discussion, however, see Bone and colleagues^36^) and/or to inform the development of novel diagnostic tools and methods for initial screening based on these essential constructs.

## Materials and Methods

### Data sample and preprocessing

The study was conducted as a part of the ASD-Net, a research network with focus on ASD that is funded by the German federal ministry of education and research^44^. All participant data came from four specialized ASD outpatient clinics in Germany where current diagnostic gold standard procedures had been applied to confirm or rule out a diagnosis of ASD. All participants were referred by specialists or self-referred to the outpatient departments. Participant data was collected retrospectively from the medical records of the respective clinic (retrospective chart review) and combined into one dataset for analysis. This procedure was approved by the Charité – Universitätsmedizin Berlin ethics committee (EA4/129/19) and due to the retrospective nature of data collection and analysis based on routinely obtained clinical data, the need for informed consent was waived by the local ethics committee. All methods were performed in accordance with the relevant institutional and international research guidelines and regulations.

The diagnostic procedure involved a standardized behavior observation in all cases (ADOS Module 4^10^), a standardized interview if parental informants were available (ADI-R^12^; care-givers were available in 62% of all cases (ASD: 71%, non-ASD: 50%)) and a differential diagnostic examination (established Structured Questionnaires and Structural Clinical Interviews frequently used in German-speaking countries), which aided trained and experienced clinicians in reaching a best-estimate clinical diagnosis. Multiple assessments were available for some of the cases, however, only the most recent assessment was considered for each case.

Our sample included data from 673 cases, of whom 57% received a diagnosis of ASD (“ASD”, n = 385) and 43% did not receive a diagnosis of ASD but relevant differential diagnoses such as affective disorders, anxiety disorders, ADHD and/or personality disorders or no current psychiatric diagnosis (“non-ASD”, n = 288; for a more detailed description of the phenotypic diversity see Supplementary Table 1). ASD subtypes according to ICD-10 (F84.0, F84.1, F84.5) were grouped together, giving us a binary outcome measure of “ASD” and “non-ASD” classes for our machine learning procedures. There was no significant difference between the two groups regarding age, gender and IQ (Table 1).

**Table 1:** Sample Description.

Characteristic	ASD (n = 385)	non-ASD (n = 288)	Statistical test
Mean Age (SD)	25.63 years (11.27)	26.81 years (12.45)	n.s. (t(582.92) = 1.27, *p* = 0.21)
% age ≥18 years [n]	67% [n = 258]	68% [n = 196]	n.s. (χ^2^(1) = 0.082, *p* = 0.78)
% age >21 years [n]	52.7% [n = 203]	51.7% [n = 149]	n.s. (χ^2^(1) = 0.065, *p* = 0.80)
Gender: % male [n]	74.3% male [n = 286]	72.9% male [n = 210]	n.s. (χ^2^(1) = 0.16, *p* = 0.69)
Mean IQ (SD)*	104.68 (16.00) (based on n = 343)	104.84 (15.49) (based on n = 245)	n.s. (t(586) = 0.12, *p* = 0.90)

Abbreviation: n.s., non significant; ASD, autism spectrum disorder; SD, standard deviation.

*Complete IQ data were available for 87% of the entire sample.

The ADOS is a standardized observation scale designed to capture important social-communicative behaviors and stereotypic and repetitive behavioral features^10^. In Module 4, which is intended for verbally fluent adolescents and adults, these aspects are coded on 31 different items. Codes fall on an ordinal scale from 0 (no abnormality related to autism) to 2 (definite evidence of abnormality) and sometimes 3 (profound severity), with additional codes of 7 and 8 for abnormal behavior or behavior not exhibited during the observation, and a code of 9 for missing values (i.e. answers omitted or left blank).

The ADOS Module 4 provides a scoring algorithm consisting of a subset of the diagnostically most informative 11 items (see Table 2) from the Social Interaction and Communication domains for calculating a comparison score, which yields an instrument classification of autism, autism-spectrum or non-spectrum.

**Table 2:** The 11 features from the ADOS Module 4 algorithm and the 5 features identified by the feature selection process for the whole sample (bold).

Code	Feature Description	ADOS core domain
A4*	Stereotyped/Idiosyncratic Use of Words or Phrases	Language/Communication
A8*	Conversation	Language/Communication
A9*	**Descriptive, Conventional, Instrumental, or Informational Gestures**	Language/Communication
A10	Emphatic or Emotional Gestures	Language/Communication
B1*	**Unusual Eye Contact**	Reciprocal Social Interaction
B2*	**Facial Expressions Directed to Others**	Reciprocal Social Interaction
B6	Empathy/Comments on Others´ Emotions	Reciprocal Social Interaction
B8	Responsibility	Reciprocal Social Interaction
B9*	Quality of Social Overtures	Reciprocal Social Interaction
B10*	**Quality of Social Response**	Reciprocal Social Interaction
B11*	**Amount of Reciprocal Social Communication**	Reciprocal Social Interaction

Abbreviation: ADOS, Autism Diagnostic Observation Scale.

*Items that are also comprised in the 12-item subset identified by Kosmicki and colleagues^41^. Further items that were identified by Kosmicki *et al*. that are not comprised in the ADOS algorithm are A7 (reporting of events), D1 (unusual sensory interest in play material/person), D2 (hand and finger and other complex mannerisms) and D4 (excessive interest in unusual or highly specific topics or objects).

For data preprocessing for our machine learning analyses, we recoded ADOS codes of 3 to 2, and codes of 7 and 8 to 0 analogue ADOS handbook. Missing values (i.e. codes of 9) were imputed using k nearest neighbor imputation with k = 5 (*knnImpute*) using the build-in *preprocess()* function form the caret R package^45^. In our dataset, six items were missing in 4–10% of all cases (items A6, B4, C1, E1, E2, E3), with all other items missing answers in less than 2.5% of the cases (for a more detailed description on the distribution of ADOS codes and missing values in our sample, see Supplementary Table 2). Furthermore, all numerical variables were normalized to range [0;1].

### Machine learning

Previous classification experiments have utilized various machine learning techniques including support vector machines, tree-based models and general linear models^32^. In these previous publications, support vector machines (SVM) were among the models that performed best^36,41,42^. Furthermore, SVM is one of the most frequently used algorithms that has been utilized for ASD classification due to its high predictive power^32^. Therefore, we decided to use SVM classification with radial kernel using svmRadial of the caret R package^45^ as our machine learning classifier. We performed an additional analysis using random forest, which showed slightly lower predictive performance. Due to readability and space constraints, we only present results for SVM. Results from our random forest analysis can be found in the supplement (see Supplementary Table 3).

All 31 ADOS items were used as features and the individuals’ best-estimate clinical diagnosis was used as our prediction class (ASD vs. non-ASD). All steps of data inspection and preprocessing, including imputation and analysis, were performed using R version 3.5.1 in Rstudio 1.1.456.

To maximize the likelihood of unbiased results, rigorous guards against overfitting were implemented. The total sample was randomly split into a 75% partition as training set for building the models and hyperparameter testing, and a 25% partition as test set for evaluation of the predictive power of the final models in completely unseen cases not used to build the model (Table 3). The random split was stratified for the outcome variable to prevent data shift between training and test set. Potential information leakage was mitigated during the missing values imputation method, i.e. information about variable distributions secretly spreading from the training set data to the test set data was avoided by performing all preprocessing steps multiple times. At each cross-validation step, preprocessing (value range normalization and *knn*-imputation) was performed separately and anew for the resampled training set folds during the model building process and once for the test set before application of the final models for prediction. The dependent variable was removed from the dataset prior to imputation to prevent information leakage. Thus, it was made sure that for imputation at each fold during cross-validation, the information about the distribution of any predictor variable in the training set was unaffected by the distribution of the same variable in the test set and of the outcome variable.

**Table 3:** Depiction of the classification task with the observed positive and negative events for the outcome in training and test set for the whole sample (“all ages”) as well as the age subgroups (“adolescents”, “adults”).

	Classification task	Training set	Test set	Total
**All ages (n** = **673)**				
Positive events	ASD	n = 289	n = 96	N = 385
Negative events	non-ASD	n = 216	n = 72	N = 288
**Adolescents ≤ 21 years (n** = **321)**				
Positive events	ASD	n = 137	n = 45	N = 182
Negative events	non-ASD	n = 105	n = 34	N = 139
**Adults** > **21 years (n** = **352)**				
Positive events	ASD	n = 153	n = 50	N = 203
Negative events	non-ASD	n = 112	n = 37	N = 149

Abbreviation: ASD, autism spectrum disorder.

Our machine learning approach consisted of multiple steps: First, we conducted a feature selection on the training set to identify a reduced feature subset with similar predictive performance profiles. We used recursive feature selection (*rfFuncs)* via the caret R package^45^ applying random forests^46^ to identify the most important features. The metric for hyperparameter tuning was Cohen’s kappa coefficient (equally weighing sensitivity and specificity), and 5-times repeated 10-fold cross-validation was applied. The second step consisted of training our reduced feature model on the training set. During model training, 5-times repeated 10-fold cross-validation was applied to mitigate the risk of overfitting and to achieve stable prediction results. The metric for hyperparameter tuning was the area under the ROC curve (AUC) used to select the model with the largest AUC on the training set. The set of tuned hyperparameters was *sigma* and *cost* with a random search of 500 different combinations. All other tunable parameters were set to default values^47^. The third step was testing our reduced feature model on the dedicated test set to see how well the reduced model can separate ASD from non-ASD in completely unseen data of a separate hold-out set, i.e. data that was never used to build the model.

To compare model performances, we evaluated the AUCs of the predictions attained from our newly identified reduced feature subset vs. all 31 items of the ADOS vs. the subset of 11 items proposed by the ADOS algorithm. Additionally, we compared model performance to the 12-item classifier previously identified by Kosmicki and colleagues^41^ in their experiments looking at children and adolescents as assessed with ADOS Module 3 (for a list of these items, see Table 2). For evaluation of the differences in terms of AUC, we relied on DeLong’s test^48^ for two correlated ROC curves as well as a bootstrap resampling significance test^49,50^ for two correlated ROC curves (each time comparing the overlap of the confidence intervals with 10 000 bootstrapped iterations).

Because of an overall large age distribution in our sample (ages ranging from 10 to 72 years, with a median age of 22 years), all of the above-mentioned steps were performed in the whole sample (“all ages”, N = 673) as well as in age subgroups of adolescents aged ≤21 years (“adolescents”, n = 321, 56.7% ASD (n = 182)) and adults aged>21 years (“adults”, n = 352, 57.7% ASD (n = 203)). For further information on the age distribution of our sample and the age subgroups, see Supplementary Table 4 and Supplementary Figures 1 to 3.

## Results

Looking at the whole sample, our recursive feature selection algorithm selected five features as the most important ones, i.e. those which had on average the highest ability to predict adolescents and adults with ASD compared to adolescents and adults with other clinically complex presentations during cross-validation: Features A9 (Descriptive, Conventional, Instrumental or Informational Gestures), B1 (Unusual Eye Contact), B2 (Facial Expressions directed to Others), B10 (Quality of Social Response) and B11 (Amount of Reciprocal Social Communication). All of the selected five features correspond to the domains of Social Interaction and Communication of the ADOS and are comprised in the original 11-feature ADOS algorithm as well as in the 12-item classifier proposed by Kosmicki and colleagues^41^ (Table 2). Items A9, B1 and B2 depict abnormalities in the participant’s reciprocal nonverbal communication and interaction observed during the ADOS examination, whereas items B10 and B11 constitute qualitative summary items, wherein the clinician rates abnormalities in the participant’s overall social behaviors during the ADOS examination.

With this reduced feature subset of only 5 features, we observed an AUC of 0.87 (sensitivity = 0.72, specificity = 0.87) in the trainings set, which is comparable to the performance of the 11-feature model of the ADOS algorithm, the 31-feature model using all items of the ADOS and the 12-item subset identified by Kosmicki and colleagues^41^ (all AUCs of 0.87; see Table 4).

**Table 4:** Performance of the machine learning models on the training and test set for the whole sample (“all ages”).

	SVM models			
	5-feature model*	11-feature model (ADOS algorithm)	31-feature model (all ADOS items)	12-feature model (Kosmicki *et al*.^41^)
**All ages (n** = **673)**				
**Training Set**				
AUC (Sensitivity, Specificity)	0.87 (0.72, 0.87)	0.87 (0.75, 0.88)	0.87 (0.73, 0.88)	0.87 (0.73., 0.85)
**Test Set**				
AUC (Sensitivity, Specificity)	0.82 (0.71, 0.83)	0.84 (0.85, 0.76)	0.84 (0.79, 0.81)	0.84 (0.77, 0.82)

Abbreviation: AUC, Area under the ROC curve; SVM, support vector machine. *5-feature model for “all ages”: A9, B1, B2, B10, B11.

**Figure 1:**
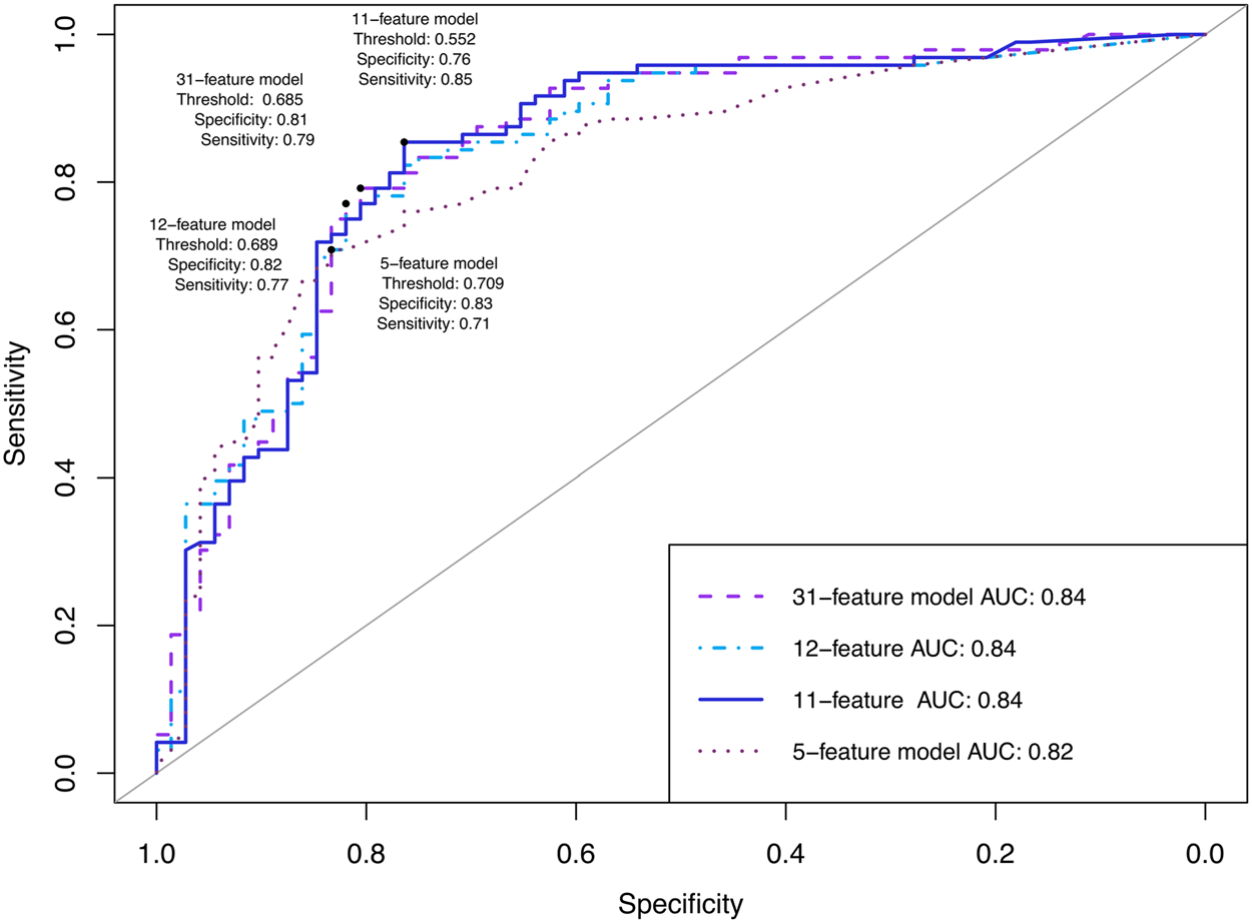
Receiver operating characteristic (ROC) curves evaluating the predictive power in the test set for the whole sample (“all ages”). Optimal ROC threshold with the highest sum of sensitivity + specificity is plotted^61^.

For independent validation of our classifiers, we computed the performance of the models on the held-out test set. When independently predicting the best-estimate clinical diagnosis, our models achieved slightly lower AUCs (see Table 4): our reduced 5-feature model achieved an AUC of 0.82 (sensitivity = 0.71, specificity = 0.83) compared to AUCs of 0.84 of the 11-feature model (sensitivity = 0.85, specificity = 0.76), the 12-feature model proposed by Kosmicki *et al*.^41^ (sensitivity = 0.77, specificity = 0.82) and the 31-feature model (sensitivity = 0.79, specificity = 0.81; Table 4, Fig. 1).

In a final step, we compared the models’ performances. No significant differences were found when comparing the AUCs of the reduced 5-feature model with the 11-feature model suggested by the ADOS algorithm (DeLong’s test: Z = −1.63, p = 0.10; bootstrapping: D = −1.61, p = 0.11, number of bootstrapped resampled = 10 000), the 12-feature model proposed by Kosmicki *et al*.^41^ (DeLong’s test: Z = −1.27, p = 0.20; bootstrapping: D = −1.26, p = 0.21, number of bootstrapped resampled = 10 000) and the 31-feature model (DeLong’s test: Z = −1.29, p = 0.20; bootstrapping: D = −1.26, p = 0.21, number of bootstrapped resamples = 10 000).

Results separately for age subgroups (“adolescents” and “adults”) can be found in Table 5. Compared to our whole sample feature subset (“all ages”: items A9, B1, B2, B10, B11), our recursive feature selection algorithm selected slightly different features as the most important ones for the specific age subgroups: items A9, B1, B2, B3, B9 for “adolescents” vs. items A9, B2, B3, B9, B10 for “adults”. Similar to the “all ages” feature subset, however, all of the selected features correspond to the domains of Social Interaction and Communication of the ADOS. Two items (B3 (Language Production and Linked Nonverbal Communication) and B9 (Quality of Social Overtures) were included in both age subgroups but not in the “all ages” sample. Item B9 is a qualitative summary item that assesses the overall quality of the individual’s attempts to initiate social interactions, whereas item B3 holds information about how the individual’s vocalizations are accompanied by nonverbal behaviors (such as eye contact, gestures and facial expression).

**Table 5:** Performance of the machine learning models on the training and test set for the age subgroups “adolescents” (≤21 years) and “adults” (>21 years).

	SVM models			
	5-feature models*	11-feature model (ADOS algorithm)	31-feature model (all ADOS items)	12-feature model (Kosmicki *et al*.^41^)
**Adolescents ≤ 21 years (n** = **321)**				
**Training Set**				
AUC (Sensitivity, Specificity)	0.83 (0.67, 0.85)	0.85 (0.58, 0.92)	0.84 (0.66, 0.85)	0.85 (0.70, 0.86)
**Test Set**				
AUC (Sensitivity, Specificity)	0.90 (0.78, 0.88)	0.88 (0.87, 0.82)	0.87 (0.84, 0.79)	0.84 (0.84, 0.77)
**Adults** > **21 years (n** = **352)**				
**Training Set**				
AUC (Sensitivity, Specificity)	0.87 (0.69, 0.88)	0.88 (0.71, 0.89)	0.86 (0.65, 0.89)	0.86 (0.62, 0.92)
**Test Set**				
AUC (Sensitivity, Specificity)	0.84 (0.90, 0.76)	0.87 (0.92, 0.84)	0.87 (0.90, 0.84)	0.85 (0.90, 0.78)

Abbreviation: AUC, Area under the ROC curve; SVM, support vector machine. *5-feature model for “adolescents”: A9, B1, B2, B3, B9. *5-feature model for “adults”: A9, B2, B3, B9, B10.

By splitting our “all ages” sample into age subgroups, we were able to increase the overall prediction accuracy of our models (see Table 5). Comparable to the whole sample, there were no significant differences between the AUCs of the 5-feature models (“adolescents”: AUC = 0.90; “adults”: AUC = 0.84), the 11-feature model proposed by the ADOS algorithm (“adolescents”: AUC = 0.88; “adults”: AUC = 0.87), the 31-feature model (“adolescents”: AUC = 0.87; “adults”: AUC = 0.87) and the 12-feature model proposed by Kosmicki *et al*.^41^ (“adolescents”: AUC = 0.84; “adults”: AUC = 0.85) in the test sets in the respective subgroups. For a detailed depiction of the comparison tests´ results as well as ROC curves for the age subgroups see Supplementary Figures 4 and 5.

## Discussion

Although the search for objective biological markers associated with ASD is currently under way, the current standard of ASD diagnosis remains based on behavioral symptoms alone. One of the most widely used behavioral examination tools which aids clinicians in reaching a best-estimate clinical diagnosis is the ADOS. Although effective, administration and evaluation of the ADOS is time-consuming and requires outstanding specific clinical expertise. However, accurate and timely identification of ASD in adulthood has become an important clinical issue^25,31^ and the field needs novel methods for initial screening as well as accurate diagnostic tools that can reach a larger percentage of cases^8^. Moreover, specificity of the ADOS in clinical settings is low, demonstrating the clinical difficulty of differentiating individuals with ASD from individuals with suspected ASD but other best-estimate psychiatric diagnoses or no psychiatric diagnoses. This is particularly challenging in high-functioning older individuals, due to symptom overlaps of various disorders with ASD, an increased comorbidity rate in ASD and the lack of reliable information about early development. Machine learning techniques have previously been applied to examine whether ASD detection can be improved by identifying core subsets of behavioral features that discriminate between children with and without ASD (ADOS Module 1 to 3). In the present study, we sought to expand on the existing literature with a specific focus on the differentiation of adolescents and adults with ASD from individuals with other clinically complex presentations (ADOS Module 4).

Using an SVM-based approach, we identified a reduced subset of 5 behavioral features from the ADOS Module 4 that showed good specificity (83%) and sensitivity (71%) on our whole sample. Furthermore, with an AUC of 82%, our reduced classifier reached performance close to that of the 11-feature algorithm proposed by the ADOS manual and the full ADOS consisting of 31 features (both AUCs of 84%) with no significant differences in overall performance. Additionally, we evaluated model performance of the previously proposed 12-item-subset by Kosmicki *et al*.^41^ on our dataset and compared performance with our models. Results showed that our reduced 5-feature model and the proposed 12-feature model (AUC of 84%) achieved similar predictive performance, with no significant differences when comparing the AUCs. In their original publication, Kosmicki *et al*.^41^ applied SVM with a radial basis function and reported model performance of 97.7% sensitivity and 97.2% specificity on their whole sample (n = 1924 ASD, n = 214 non-ASD). For individuals for whom a best-estimate clinical diagnosis was available (n = 1568 ASD, n = 175 non-ASD), their 12-feature classifier displayed 99.1% sensitivity and 70.9% specificity. Independently testing this proposed 12-item-subset on our dataset, we found an overall performance of 77% sensitivity and 82% specificity, which is lower compared to their original findings. However, their proposed 12-feature subset still achieved an overall good prediction performance that is in line with performances of our reduced subset as well as the ADOS algorithm and the full ADOS. A possible explanation for the observed lower prediction performance might lie in the composition of the investigated samples. First of all, their classifier was built using ADOS Module 3 data, with Module 3 being intended for children and adolescents. Consequently, their sample was considerably younger compared to ours and displayed an overall lower developmental level. Second, there was a large imbalance in their data between groups (ASD vs. non-ASD), with our dataset being more balanced. Lastly, while our sample was a complex clinical and naturalistic sample of individuals seen for comprehensive diagnostic evaluation, data of their investigated sample came from archival repositories and diagnosis was partly only based on ADOS results (thus missing a best-estimate clinical diagnosis). Nonetheless, our independent evaluation of their proposed classifier shows encouraging results and lends additional support to the hypothesis that models using minimal feature subsets from the ADOS are accurate for ASD classification.

By splitting up our sample further into age subgroups of adolescents (≤21 years) and adults (>21 years), we were able to even further increase prediction accuracy of our abbreviated 5-feature subsets (“adolescents”: AUC of 90%; “adults”: AUC of 84%). Similar to the whole sample (“all ages”), we found no significant differences when comparing the overall prediction performance of the different models (“adolescents”: AUCs of 90% (5-feature) vs. 88% (11-feature) vs. 87% (31-feature) vs. 84% (12-feature proposed by Kosmicki *et al*.^41^); “adults”: AUCs of 84% (5-feature) vs. 87% (11-feature) vs. 87% (31-feature) vs. 85% (12-feature proposed by Kosmicki *et al*.^41^)) in the respective subgroups.

Having a closer look at the items selected by our feature selection algorithm, we find that all 5 items identified for the whole sample (“all ages”: A9, B1, B2, B10, B11) were part of the original 11-feature algorithm proposed by the ADOS manual, thereby supporting the relative diagnostic importance of these items and possibly preserving some of its diagnostic validity. Looking more closely at the age-subgroups, results show that the majority of items overlaps between the groups (“adolescents”: A9, B1, B2, B3, B9; “adults”: A9, B2, B3, B9, B10) and that all selected items stem from the ADOS’ domains of Social Interaction and Communication. Interestingly, there is a substantial overlap of 4 items between the two age subgroups, possibly reflecting their relative importance independent of developmental level. The outstanding difference between the two age subgroups were the inclusion of the more “basic” social-communicative item B1 (Unusual Eye Contact) in the adolescent group and the more “complex” qualitative and summative item B10 (Quality of Social Response) in the adult group. These findings suggest that adolescents with ASD show more saliently unusual eye contact compared to adults with ASD whereas adults show more “complex” social interaction impairments. This finding is in line with previous studies suggesting that there may be differences in ASD symptom presentation over the lifespan due to learnt compensatory behaviors and general developmental gains^13,51^.

Comparing our findings with those of previously published minimal behavior subsets from ADOS Module 1 to 3, we find parallels in the features selected across different studies and age groups. Looking at the reduced 12-feature classifier proposed by Kosmicki and colleagues^41^ in their experiments on Module 3, we find a correspondence of all of the items identified in our classifiers apart from item B3. Regarding their experiments looking at ADOS Module 2^41^, there was an overlap of 4 items between our reduced classifiers and their identified 9-feature classifier (Gestures (A9), Unusual Eye Contact (B1), Quality of Social Overtures (B9) and Amount of Reciprocal Social Communication (B11)). Similar overlaps can be found looking at the experiments conducted by Levy and colleagues^42^, who identified reduced 5-feature classifiers for Module 2 and Module 3 with an overlap of 3 items for Module 2 (Unusual Eye Contact (B1), Facial Expressions Directed to Others (B2) and Quality of Social Response (B10)) as well as an overlap of 3 items for Module 3 (Facial Expressions Directed to Others (B2), Quality of Social Overtures (B9) and Quality of Social Response (B10)). Not yet published results of our own group also identified reduced 5-feature classifiers for Module 1, 2 and 3 using Support Vector Machine. Here, we find overlaps of feature B1 for Module 1 and features B2, B9 and B11 for Module 2 and 3 with our reduced classifier. Taken together, there are marked similarities in the features selected across different studies and age groups, particularly for items B1, B2, B9, B10 and B11, which all stem from the Social Interaction domain of the ADOS.

Impairments in social interaction in general are very common in many different psychiatric disorders. However, Bishop and colleagues^52^, for example, recently showed that the items stemming from the social interaction and communication domains of the ADOS Module 3 algorithm could be further separated into two subdomains: impairments in “Basic Social Communication” (such as eye contact, facial expression, gestures) and impairments in “Interaction Quality” including items that measure more complex aspects of social interaction (such as conversation, amount of reciprocal social communication, quality of rapport), with the former being impaired in children with ASD and mostly intact in children without ASD but other clinical diagnoses, and the latter being impaired in both groups. In line with this finding, Drimalla and colleagues^53^ for example recently developed an alternative screening method for ASD detection and showed that it was possible to accurately detect ASD compared to healthy controls by automatically analyzing the subject´s facial expression, vocal features and gaze patterns during a video-based simulated social interaction. Although all items of the ADOS algorithm focus on relevant behavioral concepts of social-communication impairments, our abbreviated classifiers might contain relevant “core” features from the ADOS that are more specific to adolescents and adults with ASD. Thus, focusing on a reduced subset of items that are most specific to ASD and less influenced by other clinical presentations could assist clinicians in the difficult process of differential diagnosis.

Our findings suggest that reduced subsets of only 5 items are equally accurate in predicting ASD as the 11-item algorithm proposed by the ADOS manual or even the whole ADOS exam consisting of 31 items. Compared to the full ADOS, however, our abbreviated 5-feature classifiers present a substantial reduction of 84% of the number of behavioral features that have to be coded in order to assess ASD. It has been argued before^39^, however, that administration time for the ADOS cannot simply be reduced by reducing the number of items coded, as ADOS items are not directly tied to any subtask. In order to code the items, it is always necessary to administer the whole-length ADOS exam (i.e. all subtasks) and consequently the time saved by coding only a smaller number of behavioral features is marginal. In contrast to this view, however, recent findings have shown that ASD classification of children can be achieved by applying the ADOS items to shorter, unstructured social interactions^54–56^ and even by relying solely on written extracts of children´s medical and educational records^57,58^, thereby suggesting that a reduction in time associated with ASD detection might be feasible. For example, Fusaro and colleagues^54^ have assessed the feasibility of applying all of the ADOS Module 1 codes, but not the behavior observation exam, to short (<10 minutes) and unstructured home-videos collected from YouTube. They collected and rated 100 videos of children (age 1–15) with self-reported ASD and non-ASD diagnoses and achieved classification accuracy of about 97% (with 94% sensitivity and 100% specificity). While certainly not all ADOS items were relevant to the YouTube videos, the authors did find, however, that a majority of the items could be applied. In particular, the items from Module 1 that overlap with the Module 4 items included in our identified 5-feature subsets (i.e. unusual eye contact (B1), facial expressions directed to others (B2), gestures (A9) and quality of social overtures (B9)) were readily detectable (frequency of “not applicable” ratings was at <0.044% per video). These findings suggest that short, unstructured interactions can provide sufficient information to rate ADOS codes and to detect ASD. Building upon this work, Tariq and colleagues^55^ recently investigated how the reduced feature subsets from the ADOS modules 1 to 3 that were identified in previous machine learning experiments^38,41,42^ could be translated into clinical practice. For this purpose, they created a mobile web portal and asked video raters to assess the previously identified minimal feature subsets in short home videos (<5 minutes) of children with and without ASD. Results showed that all video raters took a median rating time of 4 minutes to detect ASD with high accuracy (AUC at 90%). Although constrained by an unbalanced sample size, a comparison group of typically-developing children as well as relying on self-report diagnosis of ASD, these results are nonetheless promising and an important first step in translating machine-learning based behavioral models into clinical practice. Furthermore, these results support the possibility of achieving ASD detection in briefer, unstructured social interactions such as home videos of under 5- to 10-minute length, which is considerably shorter than the full ADOS exam assessment time (which is on average 30–60 minutes).

Considering these findings, the minimal 5-feature subsets identified in our study might have the potential for utility in shorter format approaches such as assessment in unstructured home videos or brief interactions with clinicians, thereby possibly reducing redundancy in the existing diagnostic algorithm and accompanying assessment time. Furthermore, the features identified could inform the development of novel diagnostic screening tools that specifically build upon these core features. Further examination is certainly needed to evaluate whether the behaviors identified with our classifiers prove essential and stable in their ability to discriminate between adolescents and adults with ASD and other complex clinical presentations and whether they may also be adequately assessed in shorter social interactions with adolescents and adults. Nonetheless, these findings support the claim that accurate ASD detection in adolescents and adults can be performed using smaller sets of behavioral features, thereby potentially allowing for a reduction in the complexity of the diagnostic procedure.

### Strengths and limitations

The main strength of this study is the large and balanced routine clinical sample of individuals all initially suspected of having ASD. The non-ASD group was a diagnostically very diverse and heterogeneous group, with ASD-like behavioral characteristics that originate, however, from different underlying psychiatric conditions such as affective disorders, anxiety disorders, ADHD and/or personality disorders or no current psychiatric diagnosis. Furthermore, there was a large age range in our sample and our sample consisted of male as well as female participants, thereby being a good representation of the routine clinical population presenting for ASD diagnostics in adolescence and adulthood. Evaluating age-restricted subgroups, we found slight differences in the items selected, hence the predictive value of our reduced algorithms might also differ in clinical as well as gender subgroups^59,60^. Future studies should investigate differences in more specific clinical comparison groups (e.g. personality disorders, anxiety disorders, other developmental disorders) as well as specific gender groups.

Additionally, our sample consisted of high-functioning individuals of whom most presented late in life for an ASD diagnosis, thereby probably belonging to the mild end of the spectrum. Therefore, results cannot be generalized to the entire ASD spectrum, especially to those individuals with lower intellectual functioning.

Lastly, our outcome criterion (best-estimate clinical diagnosis of ASD vs. non-ASD) was not independent of the features utilized for building the prediction algorithm, therefore possibly confounding our results. This circularity problem has been previously discussed, however, there are currently no ways of satisfyingly addressing this issue as there is no independent external criterion available (for a more detailed discussion see^20,41^). However, even though the ADOS was usually factored into the clinical decision making, it did not solely determine the diagnosis.

## Conclusion

Taken together, our results are an important step forward towards improving ASD detection in older individuals and shed some light particularly in the difficult issue of differential diagnosis among clinically complex cases. We identified reduced subsets of behavioral features from the ADOS Module 4 for the whole sample as well as adolescents and adults separately that showed comparable classification performance to that of the full ADOS and the existing ADOS algorithm. While all items of the ADOS focus on relevant behavioral concepts, the items identified may have a higher ability to differentiate individuals with ASD from individuals with other clinically complex presentations in adolescence and adulthood. Although further studies are needed to evaluate these reduced classifiers’ ability to generalize to completely new and unseen data and to determine its clinical value, these results may help to improve the complicated ASD diagnostic process in adolescents and adults by encouraging future efforts to improve existing diagnostic instruments such as the ADOS, thereby aiding clinicians particularly in the difficult question of differential diagnosis, as well as to develop novel diagnostic instruments for ASD detection.

## Supplementary information

Supplementary Material.

## Data Availability

The datasets generated and analysed during the current study are not publicly available due to medical confidentiality but are available from the first author on reasonable request pending the approval of the coauthors.
