# Supplementary material for: Identifying predictive features of autism spectrum disorders in a clinical sample of adolescents and adults using machine learning
Source: Sci Rep. 2020 Mar 18;10:4805. doi: 10.1038/s41598-020-61607-w (PMC7080741; doi:10.1038/s41598-020-61607-w)
Supplement: Supplementary file 1 — Supplementary Material. [file 41598_2020_61607_MOESM1_ESM.pdf]

Supplementary material for:

"Identifying predictive features of autism spectrum disorders in a clinical sample of adolescents and adults using machine learning"

Charlotte Küpper[1*], Dr. Sanna Stroth[2], Dr. Nicole Wolff[3], Florian Hauck[4], Prof. Dr. Natalia Kliewer[4], Dr. Tanja Schad-Hansjosten[5], Prof. Dr. Inge Kamp-Becker[2], Prof. Dr. Luise Poustka[6], Prof. Dr. Veit Roessner[3], Dr. Katharina Schultebraucks[7,8†], Prof. Dr. Stefan Roepke[1†]

[†]*shared last author*

Supplement 1 (Psychopathological Description):

Table S1. Psychopathological Description of Participants.

**Psychopathological description**	**ASD (n=385)**	**non-ASD (n=288)**
Other psychiatric disorders than ASD	n=160 (42%)	n=189 (66%)
ICD-10: F10-F19<br>Mental and behavioral disorders due to psychoactive substance use	n=8 (2%)	n=6 (2%)
ICD-10: F20-F29<br>Schizophrenia, schizotypal and delusional disorders	n=2 (0.6%)	n=3 (1%)
ICD-10: F30-F39<br>Affective disorders	n=88 (23%)	n=53 (18%)
ICD-10: F40-F48<br>Neurotic, stress-related and somatoform disorders	n=56 (15%)	n=61 (21%)
ICD-10: F50-F59<br>Behavioral syndromes associated with physiological disturbances and physical factors	n=2 (0.6%)	n=0 (0%)
ICD-10: F60-F69<br>Disorders of adult personality and behavior	n=15 (4%)	n=45 (16%)
ICD-10: F80-F89 *except* F84<br>Disorders of psychological development other than pervasive developmental disorders	n=15 (4%)	n=17 (6%)
ICD-10: F90-F98<br>Behavioral and emotional disorders with onset usually occurring in childhood and adolescence	n=44 (11%)	n=66 (23%)
No other psychiatric disorder	n=225 (58%)	n=99 (34%)

Abbreviations: ASD, autism spectrum disorder; ICD-10, International Statistical Classification of Diseases and Related Health Problems 10th Revision.

Supplement 2 (ADOS code distribution):

Table S2. Distribution of ADOS Module 4 codes in the ASD and non-ASD group. Code of 0 = no abnormality related to autism; Code of 1 = some abnormality; Code of 2 = definite evidence of abnormality; Code of 3 = profound severity; Code of 7 = abnormal behavior of a type that is not encompassed by the other ratings; Code of 8 = behavior not exhibited during the observation and/or the rating is inapplicable; Code of 9 = missing values (i.e. answers omitted or left blank).

		**ADOS Code**						
**ADOS Item**	**Group**	**0 n (%)**	**1 n (%)**	**2 n (%)**	**3 n (%)**	**7 n (%)**	**8 n (%)**	**9 n (%)**
**ADOS Domain: Language/Communication**								
**A1**	Non-ASD	278 (96.5)	7 (2.4)	2 (0.7)	0	-	-	1 (0.3)
	ASD	356 (92.5)	15 (3.9)	6 (1.6)	2 (0.5)	-	-	6 (1.6)
**A2**	Non-ASD	192 (66.7)	82 (28.5)	7 (2.4)	0	4 (1.4)	0	3 (1.0)
	ASD	119 (30.9)	206 (53.3)	50 (13.0)	0	1 (0.3)	1 (0.3)	8 (2.1)
**A3**	Non-ASD	287 (99.7)	0	0	0	-	-	1 (0.3)
	ASD	373 (96.9)	7 (1.8)	0	0	-	-	5 (1.3)
**A4**	Non-ASD	255 (88.5)	33 (11.5)	0	0	-	-	0
	ASD	261 (67.8)	113 (29.4)	11 (2.9)	0	-	-	0
**A5**	Non-ASD	233 (80.9)	39 (13.5)	15 (5.2)	-	-	-	1 (0.3)
	ASD	224 (58.2)	111 (28.8)	44 (11.4)	-	-	-	6 (1.6)
**A6**	Non-ASD	117 (40.6)	79 (27.4)	65 (22.6)	14 (4.9)	-	-	13 (4.5)
	ASD	69 (17.9)	108 (28.1)	137 (35.6)	55 (14.3)	-	-	16 (4.2)
**A7**	Non-ASD	205 (71.2)	63 (21.9)	12 (4.2)	3 (1.0)	-	-	5 (1.7)
	ASD	145 (37.7)	158 (41.0)	63 (16.4)	7 (1.8)	-	-	12 (3.1)
**A8**	Non-ASD	198 (68.8)	71 (24.7)	16 (5.6)	2 (0.7)	-	-	1 (0.3)
	ASD	109 (28.3)	188 (48.8)	81 (21.0)	7 (1.8)	-	-	0
**A9**	Non-ASD	211 (73.3)	51 (18.8)	13 (4.5)	7 (2.4)	-	1 (0.3)	2 (0.7)
	ASD	110 (28.6)	159 (41.3)	69 (17.9)	41 (10.6)	-	1 (0.3)	5 (1.3)

**A10**	Non-ASD	148 (51.4)	79 (27.4)	2 (0.7)	52 (18.1)	-	1 (0.3)	6 (2.1)
	ASD	68 (17.7)	130 (33.8)	34 (8.8)	139 (36.1)	-	3 (0.8)	11 (2.9)
**ADOS Domain: Reciprocal Social Interaction**								
**B1**	Non-ASD	243 (84.4)	-	43 (14.9)	-	-	-	2 (0.7)
	ASD	157 (40.8)	-	226 (58.7)	-	-	-	2 (0.5)
**B2**	Non-ASD	180 (62.5)	92 (31.9)	15 (5.2)	-	-	-	1 (0.3)
	ASD	58 (15.1)	248 (64.4)	76 (19.7)	-	-	-	3 (0.8)
**B3**	Non-ASD	186 (64.6)	39 (13.5)	1 (0.3)	-	1 (0.3)	57 (19.8)	4 (1.4)
	ASD	50 (13.0)	63 (16.4)	1 (0.3)	-	1 (0.3)	265 (68.8)	5 (1.3)
**B4**	Non-ASD	197 (68.4)	50 (17.4)	20 (6.9)	-	-	2 (0.7)	19 (6.6)
	ASD	147 (38.2)	114 (29.6)	74 (19.2)	-	-	2 (0.5)	48 (12.5)
**B5**	Non-ASD	131 (45.5)	121 (42.0)	27 (9.4)	5 (1.7)	-	-	4 (1.4)
	ASD	65 (16.9)	217 (56.4)	75 (19.5)	22 (5.7)	-	-	6 (1.6)
**B6**	Non-ASD	151 (52.4)	106 (36.8)	25 (8.7)	4 (1.4)	-	-	2 (0.7)
	ASD	67 (17.4)	185 (48.1)	100 (26.0)	32 (8.3)	-	-	1 (0.3)
**B7**	Non-ASD	171 (59.4)	79 (27.4)	27 (9.4)	7 (2.4)	-	-	4 (1.4)
	ASD	82 (21.3)	151 (39.2)	121 (31.4)	24 (6.2)	-	-	7 (1.8)
**B8**	Non-ASD	195 (67.7)	71 (24.7)	14 (4.9)	-	-	-	8 (2.8)
	ASD	202 (52.5)	122 (31.7)	56 (14.5)	-	-	-	5 (1.3)
**B9**	Non-ASD	212 (73.6)	57 (19.8)	4 (1.4)	15 (5.2)	-	-	0
	ASD	113 (29.4)	208 (54.0)	33 (8.6)	31 (8.1)	-	-	0
**B10**	Non-ASD	206 (71.5)	79 (27.4)	2 (0.7)	1 (0.3)	-	-	0
	ASD	88 (22.9)	1 (69.1)	28 (7.3)	3 (0.8)	-	-	0
**B11**	Non-ASD	168 (58.3)	98 (34.0)	16 (5.6)	4 (1.4)	-	-	2 (0.7)
	ASD	55 (14.3)	223 (57.9)	101 (26.2)	6 (1.6)	-	-	0

**B12**	Non-ASD	231 (80.2)	45 (15.6)	5 (1.7)	2 (0.7)	-	-	5 (1.7)
	ASD	187 (48.6)	156 (40.5)	28 (7.3)	5 (1.3)	-	-	9 (2.3)
**ADOS Domain: Imagination/Creativity**								
**C1**	Non-ASD	142 (49.3)	87 (30.2)	21 (7.3)	5 (1.7)	-	-	33 (11.5)
	ASD	109 (28.3)	150 (39.0)	67 (17.4)	25 (6.5)	-	-	41 (8.8)
**ADOS Domain: Stereotyped Behaviors and Restricted Interests**								
**D1**	Non-ASD	280 (97.2)	3 (1.0)	0	-	-	-	5 (1.7)
	ASD	360 (97.2)	16 (4.2)	3 (0.8)	-	-	-	6 (1.6)
**D2**	Non-ASD	276 (95.8)	2 (0.7)	3 (1.0)	-	-	-	7 (2.4)
	ASD	350 (90.9)	27 (7.0)	3 (0.8)	-	-	-	5 (1.3)
**D3**	Non-ASD	282 (97.9)	0	0	-	-	-	6 (2.1)
	ASD	376 (97.7)	2 (0.5)	0	-	-	-	7 (1.8)
**D4**	Non-ASD	253 (87.8)	26 (9.0)	2 (0.7)	0	-	-	7 (2.4)
	ASD	305 (79.2)	61 (15.8)	13 (3.4)	0	-	-	6 (1.6)
**D5**	Non-ASD	269 (93.4)	12 (4.2)	1 (0.3)	-	-	-	6 (2.1)
	ASD	330 (85.7)	42 (10.9)	3 (0.8)	-	-	-	10 (2.6)
**ADOS Domain: Other abnormal behaviors**								
**E1**	Non-ASD	258 (89.6)	12 (4.2)	1 (0.3)	-	1 (0.3)	-	16 (5.6)
	ASD	354 (91.9)	14 (3.6)	0	-	4 (1.0)	-	13 (3.4)
**E2**	Non-ASD	260 (90.3)	11 (3.8)	1 (0.3)	-	-	-	16 (5.6)
	ASD	358 (93.0)	11 (2.9)	0	-	-	-	16 (4.2)
**E3**	Non-ASD	213 (74.0)	50 (17.4)	9 (3.1)	-	-	-	16 (5.6)
	ASD	287 (74.5)	74 (19.2)	8 (2.1)	-	-	-	16 (4.2)

Abbreviations: ASD, autism spectrum disorder; ADOS, Autism Diagnostic Observation Scale.

Supplement 3 (random forest models):

For random forest models, we used 'rf' R package in caret. We used 500 fully grown random forests with the parameter "minimum node size" fixed at 1 (default for classification) to fine-tune the number of randomly selected predictors per split ('mtry') using cross-validation. All other hyperparameters were set to default values.

Table S3. Performance of the machine learning models on the training and test set using random forest.

	**Random Forest Models**			
	**5-feature model***	**11-feature model (ADOS algorithm)**	**12-feature model (Kosmicki et al. [41])**	**All-feature model (all ADOS items)**
**Training Set** AUC (Sensitivity, Specificity)	.85 (.72, .81)	.88 (.76, .86)	.87 (.77., .86)	.88 (.77., .86)
**Test Set** AUC (Sensitivity, Specificity)	.80 (.76, .75)	.84 (.79, .79)	.83 (.79, .78)	.84 (.74, .85)

*5-feature model for "all ages": items A9, B1, B2, B10, B11.

Abbreviation: AUC, Area under the ROC curve; RF, random forest.

Supplement 4 (age distribution of the whole sample and the age subgroups):

Figure S1. Age frequency distribution in the whole sample (“all ages”).

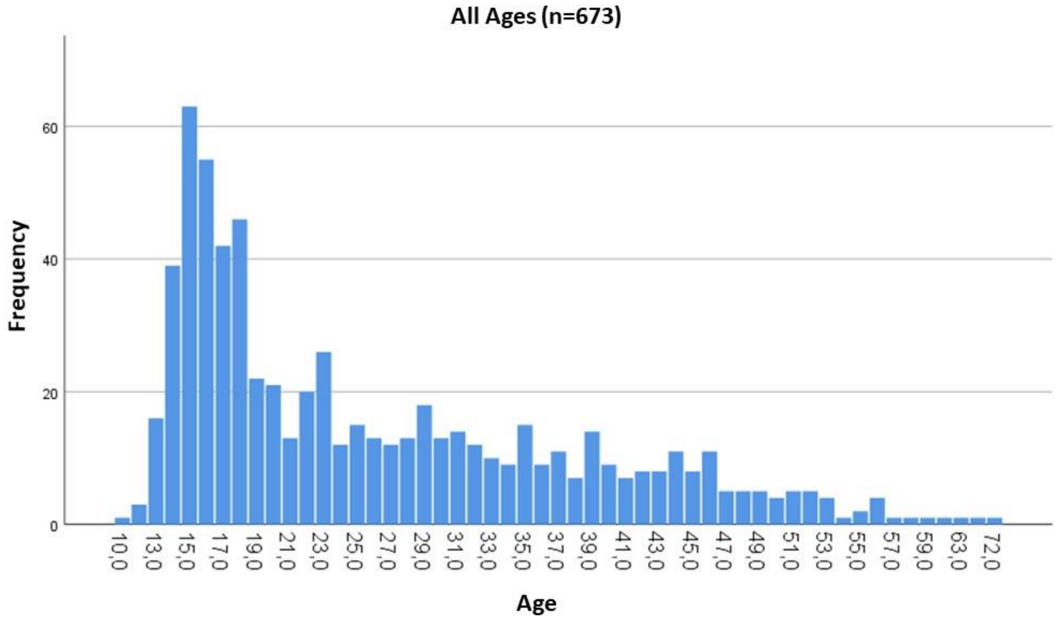

Table S4. Sample Description of the age subgroups (“adolescents”, “adults”).

**Characteristic**	**Adolescents ≤ 21 years (n=321)**	**Adults > 21 years (n=352)**
ASD: % ASD diagnosis [n]	56,7% ASD [n=182]	57,7% ASD [n=203]
Mean Age (SD)	16.42 years (2.14)	34.97 years (9.90)
Median Age	16 years	33 years
Gender: % male [n]	81.3% male [n=261]	66.8% male [n=235]

Abbreviation: ASD, autism spectrum disorder; SD, standard deviation.

Figure S2. Age frequency distribution in the “adolescents” subgroup.

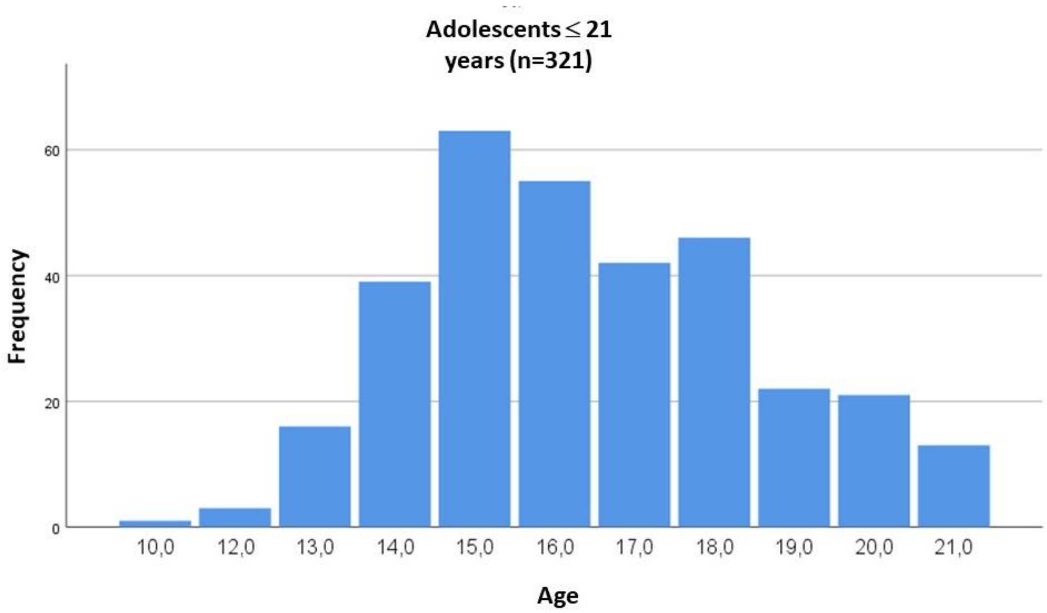

Figure S3. Age frequency distribution in the “adults” subgroup.

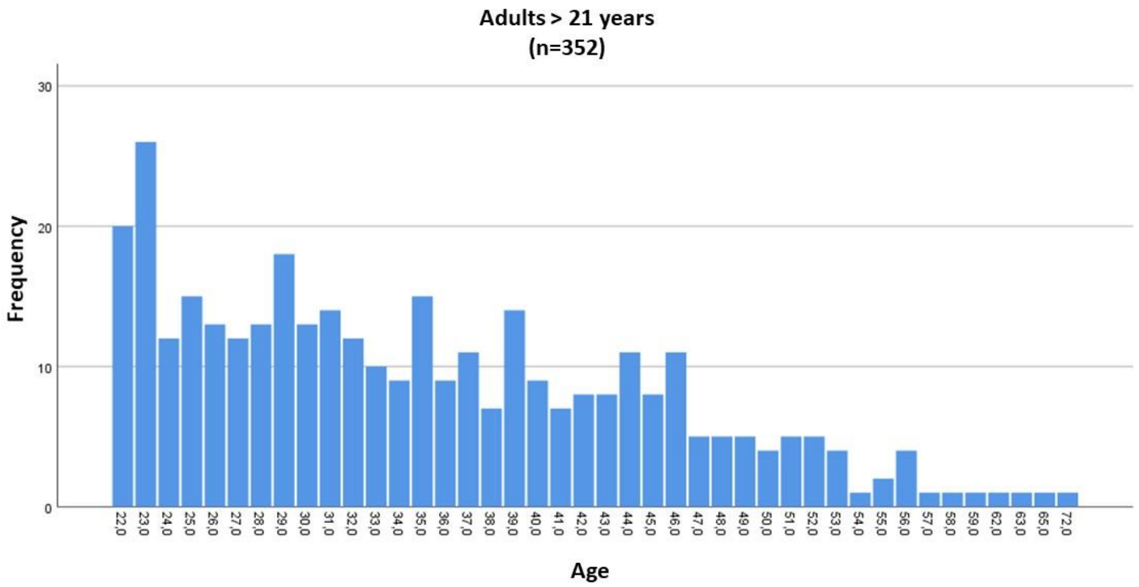

Supplement 5 (ROC curves of the age subgroups ("adolescents" and "adults"):

Figure S4. Receiver operating characteristic (ROC) curves for the "adolescents" subgroup evaluating the predictive power in the test set. Optimal ROC threshold with the highest sum of sensitivity + specificity is plotted.

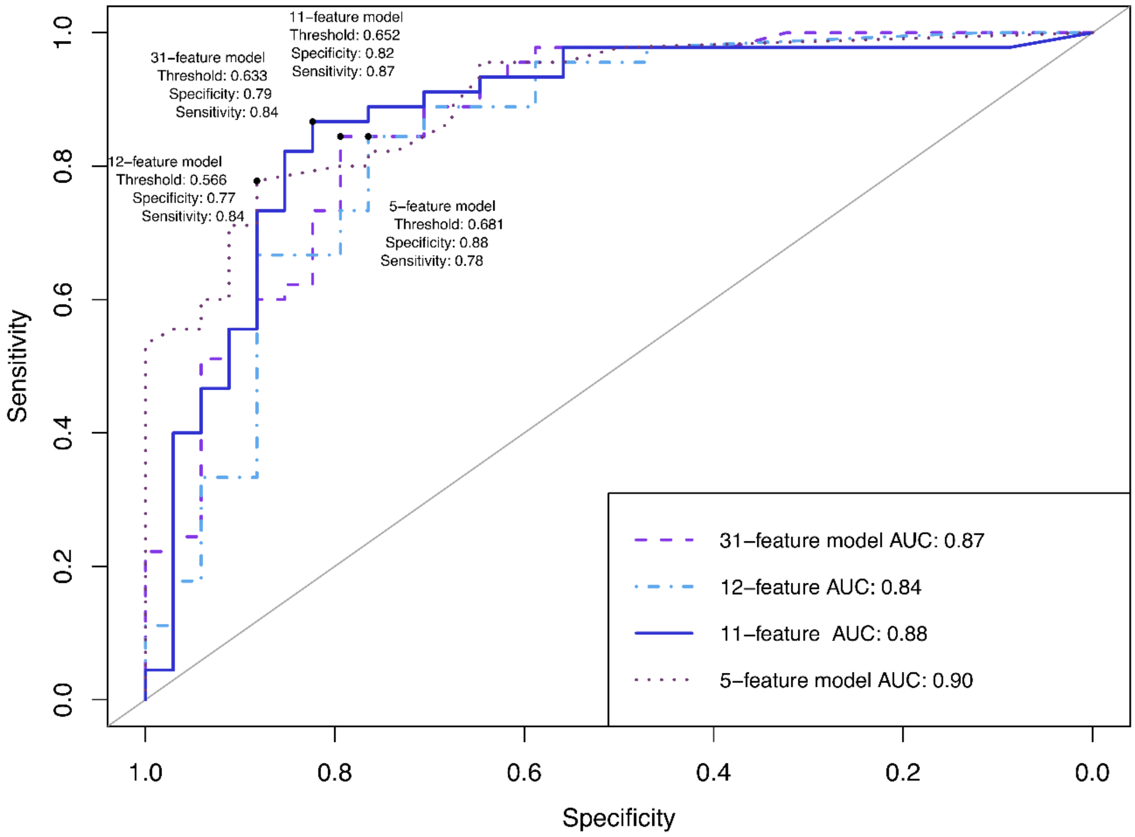

"Adolescents" subgroup: No significant differences were found when comparing the AUCs of the reduced 5-feature model with the 11-feature model suggested by the ADOS algorithm (DeLong's test: Z = 0.85, p = .40; bootstrapping: D = 0.85, p =.39, number of bootstrapped resampled = 10 000), the 12-feature model proposed by Kosmicki et al. [41] (DeLong's test: Z = 1.49, p = .14; bootstrapping: D = 1.48, p =.14, number of bootstrapped resampled = 10 000) and the all-feature model (DeLong's test: Z = 0.94, p = .35; bootstrapping: D = 0.93, p =.35, number of bootstrapped resamples = 10 000).

Figure S5. Receiver operating characteristic (ROC) curves for the "adults" subgroup evaluating the predictive power in the test set. Optimal ROC threshold with the highest sum of sensitivity + specificity is plotted.

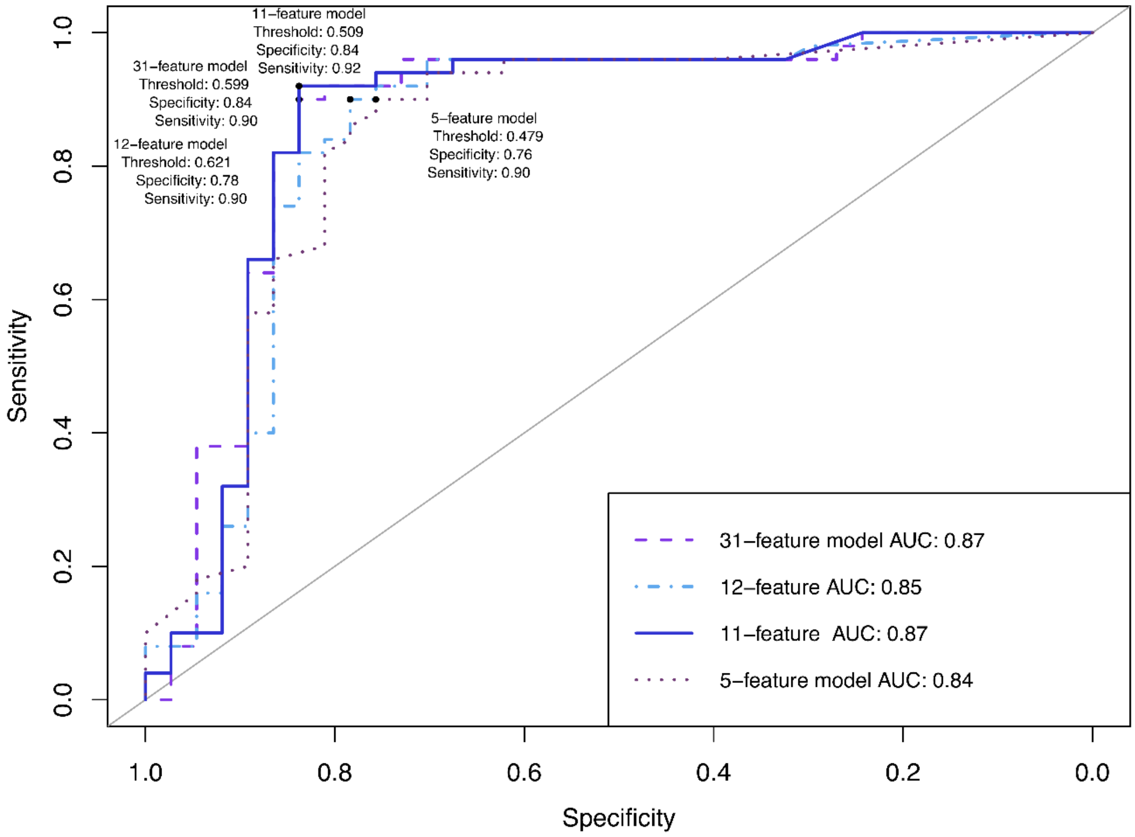

<u>"Adults" Subgroup:</u> No significant differences were found when comparing the AUCs of the reduced 5-feature model with the 11-feature model suggested by the ADOS algorithm (DeLong's test: Z = -1.60, p = .11; bootstrapping: D = -1.54, p =.12, number of bootstrapped resampled = 10 000), the 12-feature model proposed by Kosmicki et al. [41] (DeLong's test: Z = -0.30, p = .76; bootstrapping: D = -0.30, p =.76, number of bootstrapped resampled = 10 000) and the all-feature model (DeLong's test: Z = -1.76 , p = .08; bootstrapping: D = -1.71, p =.09, number of bootstrapped resamples = 10 000).
